# Spin polarized nodal loop state at Fermi level in the monolayer PrClS

**DOI:** 10.3389/fchem.2025.1544147

**Published:** 2025-02-27

**Authors:** Yilin Zhao, Li Zhang, Yufeng Gao

**Affiliations:** ^1^ School of Science, Beihua University, Jilin, China; ^2^ College of Mechanics, Changchun Institute of Technology, Changchun, China; ^3^ School of Mathematics, Jilin University, Changchun, China; ^4^ College of Mathematics, Tonghua Normal University, Tonghua, China

**Keywords:** nodal loop state, topological state, half metallicity, spin polarization, monolayer magnet

## Abstract

The investigation of two-dimensional materials exhibiting half-metallicity and topological features has become a rapidly growing area of interest, driven by their immense potential in nanoscale spintronics and quantum electronics. In this work, we present a comprehensive study of a two-dimensional PrClS monolayer, revealing its remarkable electronic and mechanical properties. Under its ferromagnetic ground state, the PrClS monolayer is shown to exhibit half-metallic behavior with 100% spin polarization originating from the spin-up channel. Of particular significance is the discovery of a spin-polarized nodal loop state within the spin-up channel. This intriguing state, characterized by a critical dispersion type and its precise alignment with the Fermi energy level, represents a feature of great interest for practical spintronic and quantum applications. Further analysis of the nodal loop topology using a maximally localized Wannier tight-binding Hamiltonian unveils distinct topological edge states. These edge states emerge clearly from the nodal loop crossings and are entirely separated from the bulk band projection, ensuring enhanced experimental detectability. The robustness of this nodal loop state is also explored under the influence of spin-orbit coupling, where it transforms into a unique hourglass-shaped dispersion while maintaining its fundamental characteristics, further solidifying its potential for experimental validation and deployment in advanced technologies. To assess the applicability of the PrClS monolayer in practical settings, its mechanical properties were thoroughly evaluated and several key parameters were analyzed, revealing significant mechanical anisotropy. This anisotropy underscores the importance of directional dependence in structural engineering and highlights the material’s versatility for applications requiring tailored mechanical responses. Overall, the PrClS monolayer represents an exceptional platform for investigating spin-polarized topological phenomena and demonstrates strong potential as an exciting material for both fundamental research and technological innovation.

## Introduction

The investigation of topological states has emerged as a cornerstone of modern condensed matter physics and solid-state material science (Zhang T. et al., 2019; Tang et al., 2019a; Tang et al., 2019b; Xiao and Yan, 2021; Narang et al., 2021), driven by groundbreaking developments in topological band theory (Bansil et al., 2016; Chiu et al., 2016; Hasan and Kane, 2010; Qi and Zhang, 2011). This theoretical framework has revolutionized our understanding of topological phenomena in crystalline materials, offering a systematic approach to link structural symmetries with the constraints imposed by band topology (Vergniory et al., 2019; Bradlyn et al., 2017). Initially focused on topological insulators, research in this domain has expanded significantly to include a wide array of systems, such as topological semimetals (Yu C. et al., 2021; Soluyanov et al., 2015; Burkov, 2016; Yan and Felser, 2017; Gao et al., 2019; Burkov, 2018; Schoop et al., 2018; Weng et al., 2016; Bernevig et al., 2018; Hirayama et al., 2018; Armitage et al., 2018; Xu, 2020; Li et al., 2020; Li and Xia, 2020; Xu et al., 2020), as well as quasiparticles like topological photons (Liu et al., 2021; Yang et al., 2018; Pan et al., 2023; Lin et al., 2023; Hu et al., 2022; Deng et al., 2022), magnons (Li et al., 2017; Nie et al., 2020; Zhu et al., 2021; Corticelli et al., 2022; Moghaddam et al., 2022; Gordon et al., 2021; He B. et al., 2021) and phonons (Yang Y. et al., 2019; Long et al., 2020; Wang et al., 2020; Xia et al., 2019; Yang, 2022a; Li, 2023; Yang, 2022b). Each of these systems features topological quasiparticles characterized by diverse pseudospin textures, dispersion behaviors, and topological charges, alongside intricate topological manifolds (Yu Z. et al., 2021). A hallmark of topological materials is the presence of nontrivial surface states, which serve as critical signatures of their topological nature (Moore, 2010; Kim et al., 2012; Zhang et al., 2009; Kargarian et al., 2016). These surface states not only validate theoretical predictions but also play a pivotal role in experimental investigations (Xu et al., 2015; Zhang T. T. et al., 2019; Xiao et al., 2020; Yu et al., 2017; Zhang et al., 2020; Takane et al., 2019; Yang et al., 2023). Their origin lies in the interplay between symmetry operations and the crystal space group symmetries of a given material. For example, topological nodal points give rise to Fermi arc surface states, while drumhead surface states are associated with nodal lines or loops where crossing regions define their boundaries. These unique surface phenomena not only highlight the fundamental characteristics of topological materials but also provide a platform for exploring novel quantum phenomena. The implications of these nontrivial surface states extend far beyond fundamental physics, offering transformative potential for next-generation quantum technologies. The exceptional electronic, optical, and thermal properties of topological materials position them as promising candidates for the development of advanced quantum devices and innovative technological applications. As the field continues to evolve, the exploration of these properties opens new avenues for leveraging topological materials in cutting-edge technological breakthroughs. By broadening the scope to encompass diverse systems and quasiparticles, the study of topological states offers profound insights into the interplay between symmetry, topology, and material properties. This interdisciplinary approach not only deepens our understanding of the underlying physics but also paves the way for translating these discoveries into practical, real-world applications.

During recent years, the exploration of topological states in two-dimensional (2D) materials has gained tremendous momentum, offering a distinct perspective that complements the extensive studies conducted on their three-dimensional (3D) counterparts (Miró et al., 2014; Chen et al., 2024; Zhong et al., 2024; Xie et al., 2023; Li et al., 2023; Liu et al., 2023; Zhong et al., 2023; Yu et al., 2023; Guo P.-J. et al., 2023; Zhang et al., 2023). From a practical standpoint, 2D materials present several inherent advantages, including seamless structural integration, compatibility with existing technological platforms, and straightforward incorporation into device architectures. These features position 2D materials as highly promising candidates for a diverse range of technological applications, from quantum devices to spintronic systems. Despite their reduced dimensionality, 2D materials are capable of exhibiting topological behaviors that parallel those observed in 3D bulk materials. Specifically, topological states in 2D systems are characterized by features such as band degeneracy, dispersion profiles, and the spatial organization of band crossings. However, a key distinction arises in 2D materials due to the transition from the surface states commonly observed in 3D systems to edge states, which dominate in reduced dimensions. This transformation provides a unique platform for studying the interplay between topology and dimensionality, particularly in systems influenced by magnetic interactions. A particularly noteworthy area of focus is 2D half-metallic materials that combine nontrivial topological band structures with 100% spin polarization in their conductive channels (Jia et al., 2020; Zhao and Wang, 2020; Sun and Luo, 2022; Guo S.-D. et al., 2023; Zhang et al., 2024; Su et al., 2023; Yang T. et al., 2019). These materials represent a significant breakthrough in the field, as they enable the emergence of spin-polarized topological edge states. Even more compelling is the dynamic tunability of these states, which can be controlled by altering the direction of the material’s magnetization. This added degree of control, absent in nonmagnetic 2D counterparts, introduces exciting possibilities for device design and experimental manipulation. The ability to manipulate spin-polarized edge states on demand offers substantial advantages for the development of next-generation quantum devices and spintronic applications. Magnetic 2D materials further distinguish themselves by offering a synergistic interplay between topology and spintronic functionality. In contrast to nonmagnetic systems, the magnetic nature of these materials introduces a straightforward mechanism for tuning their topological properties, providing a highly effective framework for engineering versatile and adaptable nanoscale devices. This degree of control, combined with their technological compatibility and the simplicity of incorporating them into existing fabrication processes, underscores their transformative potential in both fundamental research and practical applications.

Despite significant progress in the study of magnetic 2D topological materials, the field continues to evolve as a dynamic frontier in condensed matter physics. A critical challenge remains the limited pool of magnetic 2D materials compared to their 3D counterparts, particularly those with robust, tunable, and highly stable topological properties. This disparity underscores the urgent need for targeted efforts in the discovery, synthesis, and characterization of novel magnetic 2D materials optimized for advanced topological configurations. Addressing these challenges will deepen our understanding of the intricate interplay between dimensionality, magnetism, and topology, while simultaneously paving the way for transformative advancements in quantum technologies and spintronic applications. In this study, we identify monolayer praseodymium chloride sulfide (PrClS) as a promising candidate material with extraordinary topological characteristics. Through comprehensive theoretical analysis and effective model calculations, we demonstrate that this magnetic 2D compound exhibits half-metallic behavior under its ferromagnetic ground state. Most notably, in the conducting spin-up channel, we uncover the existence of a spin polarized topological nodal loop state that is precisely aligned with the Fermi energy level and exhibits a critical dispersion type, making it highly appealing for spintronic and quantum applications. The symmetry properties and detailed mechanisms underlying its band dispersion have been thoroughly determined, offering insight into the material’s unique electronic structure. Further exploration reveals the topological edge spectrum of the PrClS monolayer are clearly separated from the bulk bands, enhancing their experimental detectability. Upon incorporating the effects of spin-orbit coupling (SOC), we find that while the nodal loop persists, its band dispersion transforms into an intriguing hourglass shape, further highlighting the robustness and exotic nature of the material’s topological features. To evaluate the material’s potential for practical applications, we have also analyzed its mechanical properties, deriving key parameters such as Young’s modulus, shear modulus, and Poisson’s ratio, and investigating their anisotropic behaviors. In conclusion, this work introduces the PrClS monolayer as an exceptional magnetic 2D material that synergizes half-metallicity, spin-polarized nodal loop states, and robust topological properties. Its unique combination of characteristics establishes PrClS as an ideal platform for exploring fundamental physics in 2D magnetic systems, while also offering significant potential for the development of next-generation quantum technologies and spintronic devices.

## Computational methology

First-principles calculations were performed within the framework of density functional theory (DFT) (Rajagopal and Callaway, 1973; Kohn and Sham, 1965) using the Vienna Ab initio Simulation Package (VASP) (Kresse and Furthmüller, 1996; Hafner, 2008) and the projected augmented wave (PAW) method (Blöchl, 1994; Kresse and Joubert, 1999). Exchange-correlation interactions were treated using the Perdew–Burke–Ernzerhof (PBE) functional under the generalized gradient approximation (GGA) framework (Perdew et al., 1996). The computational parameters included a plane-wave energy cutoff of 520 eV and an 8 × 8 × 1 Monkhorst–Pack k-point grid for sampling the first Brillouin zone (Monkhorst and Pack, 1976). Valence electron configurations were explicitly defined as Pr (4 f^2^5s^2^5p^6^5d^1^6s^2^), Cl (3s^2^3p^5^) and S (3s^2^3p^4^). A vacuum layer of 17 Å was introduced in the simulation box to prevent interactions between periodic images. Long-range van der Waals forces were accounted for using the DFT-D3 correction method (Grimme et al., 2010; Grimme et al., 2011). Structural optimization and self-consistent calculations were performed with convergence criteria set to a maximum atomic force of less than 1 × 10^−3^ eV/Å and a total energy change below 1 × 10^−6^ eV per atom. Given the strong correlation effects associated with the lanthanide element Pr, we have employed the strongly constrained and appropriately normed (SCAN) meta-GGA functional (Sun et al., 2015) to analyze the electronic band structure. This method has been shown to match or even enhance the accuracy of computationally intensive hybrid functionals while operating at nearly GGA cost (Sun et al., 2016). Phonon dispersion relations were computed via the finite-displacement method combined with density functional perturbation theory (Refson et al., 2006; Togo and Tanaka, 2015), as implemented in the Phonopy package (Togo et al., 2023; Togo, 2022). To evaluate thermodynamic stability, *ab initio* molecular dynamics (AIMD) simulations were conducted on a 4 × 4 × 1 supercell over 5 picoseconds at temperatures ranging from 100 K to 300 K within a canonical NVT ensemble (Payne et al., 1992; Bucher et al., 2011). Post-processing and data visualization were streamlined using the VASPKIT high-throughput toolkit (Wang et al., 2021). Mechanical properties were analyzed by extracting the stress-strain relationship (Wang et al., 1995). To investigate the system’s topological properties, maximally localized Wannier functions were constructed using the WANNIER90 package (Mostofi et al., 2008; Mostofi et al., 2014), followed by the computation of projected surface states with the WANNIERTOOLS software (Wu et al., 2018). These comprehensive methodologies provide a robust foundation for understanding the structural, mechanical, and topological characteristics of the system.

## Results and discussions

**FIGURE 1:**
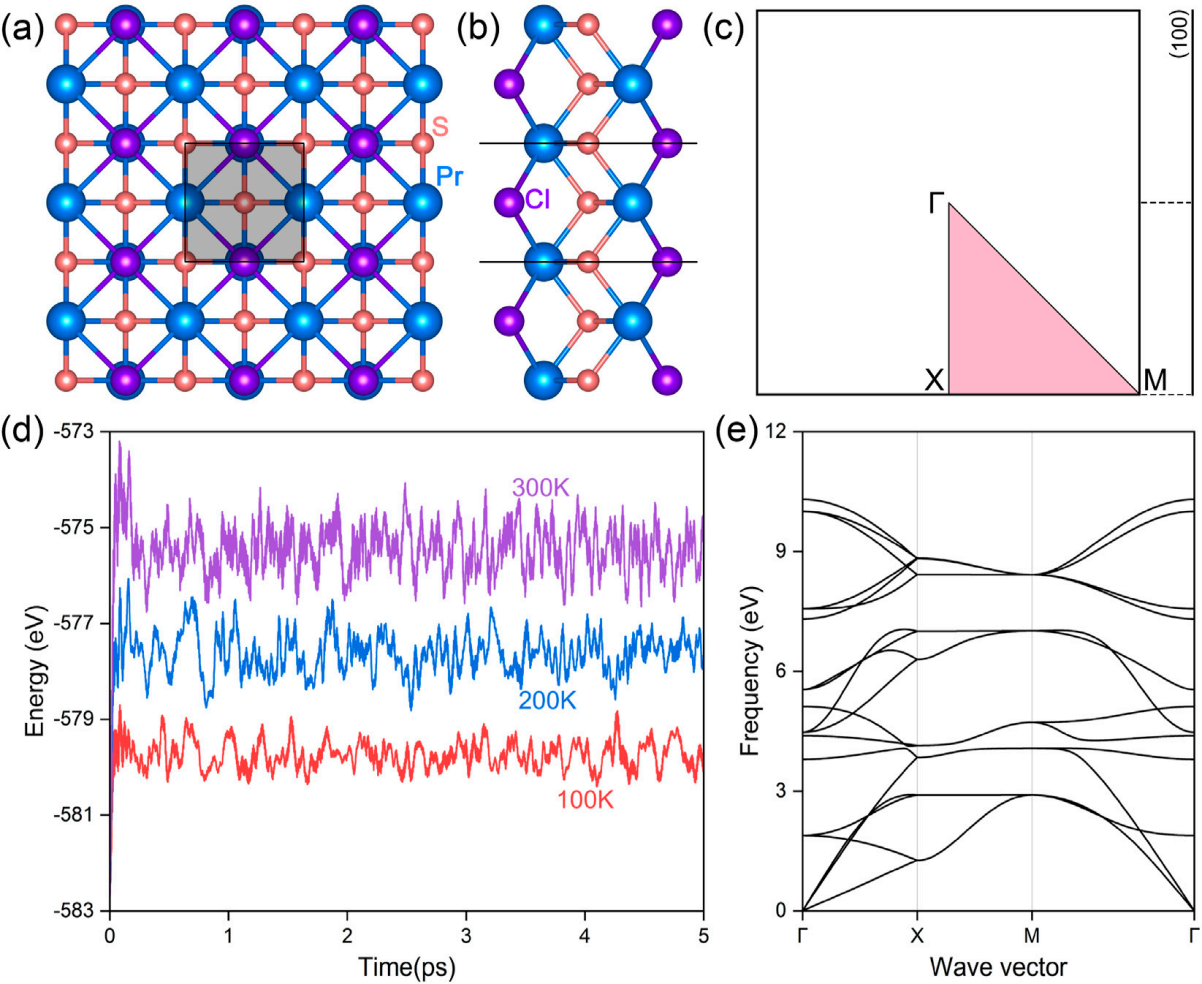
**(A)** Top and **(B)** side views of the monolayer PrClS, with atoms represented by spheres of different colors as indicated by the label. **(C)** The corresponding two-dimensional Brillouin zone and the (100) projected edge, highlighting the high-symmetry points and paths. Total potential energy fluctuations **(D)** and the phonon spectrum **(E)** of the PrClS monolayer. Ab initio molecular dynamics (AIMD) simulations were conducted for 5 picoseconds at temperatures of 100 K, 200 K, and 300 K.

The praseodymium chloride sulfide (PrClS) monolayer was initially screen out as a van der Waals (vdW) material from Materials Cloud (Mounet et al., 2018) and it adopts a 2D tetragonal crystal structure, belonging to the P4/nmm space group (No. 129). Figures 1A, B illustrate the structural arrangement, providing the top and side views of the lattice, respectively. In the top view, the shaded area marks the primitive cell, comprising two praseodymium (Pr) atoms, two chlorine (Cl) atoms, and two sulfur (S) atoms, occupying the 2c, 2c, and 2b Wyckoff positions, respectively. Due to the unpaired electron configuration of the lanthanide Pr atom, we analyzed both ferromagnetic and antiferromagnetic states to determine the system’s magnetic ground state. We utilized a 2 × 2 supercell model and examined various magnetic configurations, including one ferromagnetic (FM) state and four antiferromagnetic (AFM) configurations, as illustrated in Supplementary Figure S1 of supplementary materials. Total energy calculations using both PBE-GGA and SCAN meta-GGA consistently indicated that the ferromagnetic configuration has the lowest total energy. The calculations indicate that the ferromagnetic state corresponds to the stable magnetic configuration of the PrClS monolayer. A closer examination of the side view reveals a quintuple-layer stacking sequence, arranged vertically as Cl–Pr–S–Pr–Cl. The lattice constant was optimized to a = b = 4.374 Å, consistent with the tetragonal symmetry of the system. Within the lattice, the bond lengths were calculated as 2.731 Å for Pr–S and 3.344 Å for Pr–Cl. The bond angles exhibit structural anisotropy, with the Pr–S–Pr bond angle measuring 106.41°, significantly larger than the Pr–Cl–Pr bond angle of 81.69°. These geometric features highlight the unique structural and magnetic properties of the PrClS monolayer.

The PrClS monolayer’s distinctive bonding environment and inherent magnetic properties highlight its potential for applications in 2D materials and magnetism-focused research. Although the structural configuration of the PrClS monolayer was initially cataloged in the C2DB database (Gjerding et al., 2021; Haastrup et al., 2018), further investigations were necessary to evaluate its thermal and dynamic stability comprehensively. To assess its thermal robustness, AIMD simulations were conducted using a 4 × 4 × 1 supercell at temperatures of 100 K, 200 K, and 300 K. These simulations spanned 5,000 time steps, with each step corresponding to 1 femtosecond. As shown in Figure 1D, the total energy of the PrClS monolayer exhibited minimal fluctuations during the simulation, and the structural integrity was maintained without noticeable distortions across all tested temperatures. This result demonstrates the monolayer’s exceptional thermal stability, even at temperatures up to 300 K. To complement the thermal analysis, the phonon dispersion of the PrClS monolayer was calculated to examine its dynamic stability. The phonon spectrum, illustrated in Figure 1E, reveals the absence of soft modes, confirming its dynamic stability. Together, these findings provide compelling evidence of the PrClS monolayer’s stability under both thermal and dynamic conditions, suggesting its feasibility for experimental synthesis. In addition to this remarkable stability, the thermal stability of magnetic ordering is crucial, particularly in the face of thermal fluctuations at and above room temperature. To assess this, we conducted a Monte Carlo simulation to estimate the Curie temperature, with detailed calculation parameters provided in the supplementary materials. Our results indicate that the Curie temperature of the PrClS monolayer is 416.85 K, see Supplementary Figure S2 of the supplementary materials. This exceptionally high Curie temperature, which exceeds room temperature, underscores the potential of PrClS for spintronic applications that operate above room temperature. The demonstrated stability of the PrClS monolayer lays a strong foundation for its integration into various practical applications. These results position it as a highly promising candidate for further study and potential deployment in advanced 2D material systems.

**FIGURE 2:**
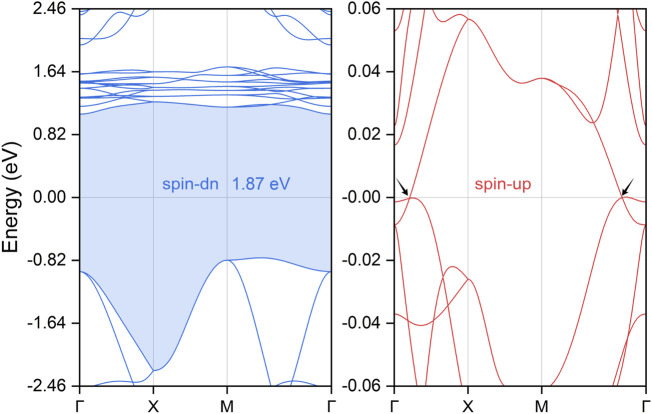
Electronic band structures of the PrClS monolayer. The spin-down band structure exhibits an insulating character with a band gap of 1.87 eV, while the spin-up band structure displays metallic behavior, featuring two band crossing points at the Fermi level, as indicated by the black arrows.

**FIGURE 3:**
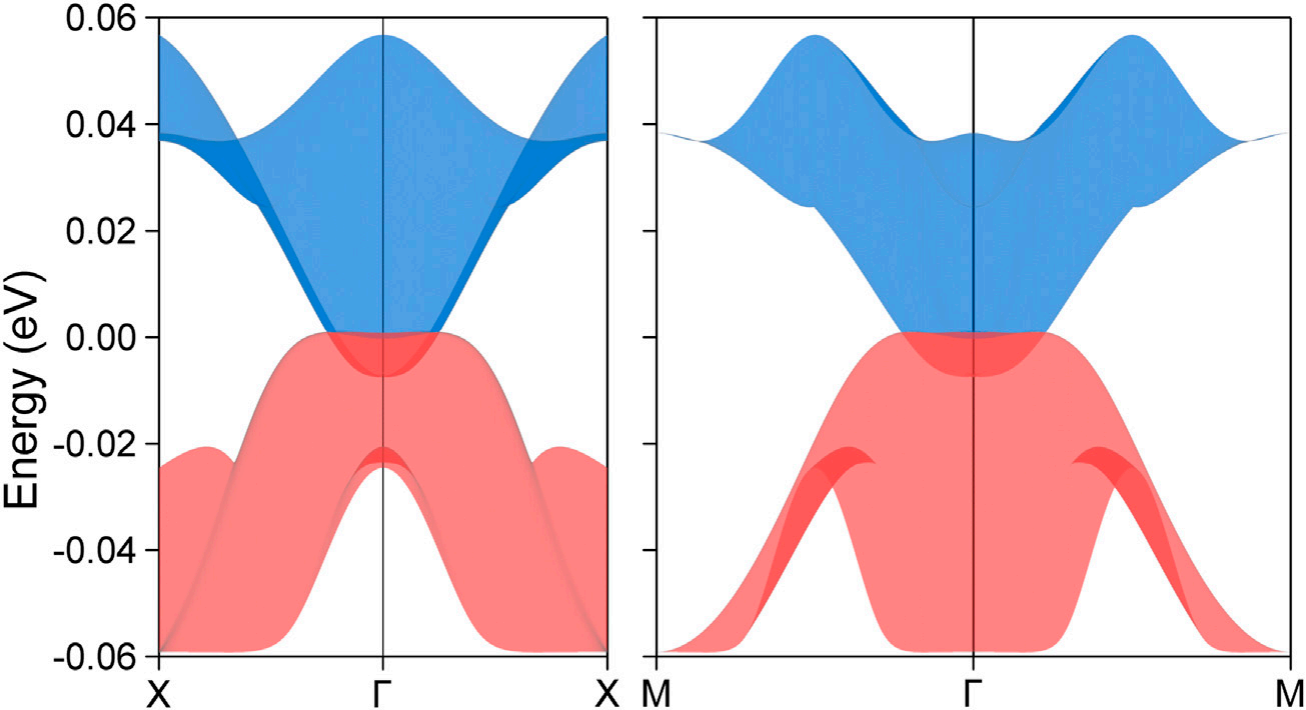
The calculated three-dimensional band structure dispersions for the PrClS monolayer, projected along the high-symmetry paths X–Γ–X and M–Γ–M. The consistent color scheme has been employed across the band surfaces to facilitate comparison between different projection paths.

The spin-polarized electronic band structures of the PrClS monolayer, based on its optimized crystal structure, were calculated and are presented in Figure 2. The Fermi energy level is set as the reference point at 0 eV. High-symmetry k-paths for the calculations were determined using the crystallographic parameters of the structure and identified with the Seek-Path code (Hinuma et al., 2017), as illustrated in Figure 1C. In this analysis, SOC effects were excluded; these will be addressed in detail in later sections. The computed band structures, displayed in Figure 2, reveal that the PrClS monolayer exhibits distinct half-metallic behavior, characterized by pronounced spin splitting between the spin channels. The left panel of Figure 3, corresponding to the spin-down channel, indicates an insulating state with a band gap of 1.87 eV. Conversely, the right panel, representing the spin-up channel, shows multiple bands crossing the Fermi level, confirming its metallic nature. This unique half-metallic property results in 100% spin polarization of the conducting electrons, positioning the PrClS monolayer as a highly promising material for spintronic applications. To further explore the mechanism of such half-metallicity, we have performed detailed analyses of the total density of states (DOS) and projected density of states (PDOS) at the low-energy electronic states. As illustrated in the following Supplementary Figure S3 of the supplementary materials, the total DOS reveals that the low-energy states near the Fermi level are predominantly contributed by the Pr atom. More importantly, these states exhibit a significant spin-splitting effect, particularly evident from the two asymmetric peaks in the energy range of 0–2 eV between the two spin channels. Further decomposition using the projected DOS indicates that the *f* orbitals of the Pr element are primarily responsible for this strong spin-splitting. This orbital-driven spin polarization ultimately leads to the half-metallic behavior in the band structures. Of particular significance within the metallic spin-up channel are two band-crossing points observed along the Γ–X and M–Γ high-symmetry paths, as indicated by black arrows in Figure 2. These crossings occur precisely at the Fermi level. Upon examining their dispersive features, these points are identified as critical type, lying between type-I and type-II states (He T. et al., 2021; Jin et al., 2019; Yang et al., 2020; Yang et al., 2021; Zhang and Wang, 2020; Li et al., 2022). This intermediate classification suggests unique electronic properties, offering valuable opportunities for future exploration in advanced electronic and spintronic devices (Yu C. et al., 2021; Xu, 2020). To account for the strong correlation effects in the lanthanide Pr, we employed the SCAN meta-GGA functional to further evaluate the electronic band structure of the PrClS monolayer. The calculated results, presented in Supplementary Figure S4 of the supplementary materials, demonstrate that the half-metallic behavior and the band crossing condition are both adequately preserved, especially for the energy level and dispersion type.

Focusing exclusively on the single conducting spin-up channel, the PrClS monolayer, which crystallizes in the tetragonal P4/nmm space group, displays a rich array of symmetry operations. These include fourfold rotation *C*_4z_, twofold rotation *C*_2z_, inversion symmetry *P*, and time-reversal symmetry *Τ*. Collectively, these symmetries define the D_4h_ little group at the Γ point within the Brillouin zone. A direct consequence of these symmetry properties is that the two band-crossing points along the Γ–X and M–Γ paths are not isolated phenomena but rather components of a continuous nodal loop encircling the Γ point within the 2D Brillouin zone. Nodal loops typically exhibit finite energy variations and diverse dispersion relationships along their paths. However, a distinctive feature of the PrClS monolayer’s band structure is that the two crossing points along the Γ–X and M–Γ directions are precisely aligned with the Fermi energy, as illustrated by the horizontal reference line in the right panel of Figure 2. The observed alignment highlights the material’s remarkable symmetry and points to its unconventional electronic properties. Although symmetry analysis verifies the presence of the nodal loop, it falls short in fully capturing the intricate energy variations and dispersion profiles along its trajectory. To gain a more comprehensive understanding, an in-depth analysis of the band structure was performed specifically within the *k*_z_ = 0 plane. This investigation yielded 3D band dispersion plots, which are presented in Figure 3. To enhance interpretability, two distinct projection paths, X–Γ–X and M–Γ–M, were examined, with consistent color schemes applied to represent the band surfaces. These visual representations unambiguously reveal that the two crossing points constitute a closed nodal loop. This finding not only corroborates the symmetry-based predictions but also provides a more nuanced understanding of the electronic behavior of the PrClS monolayer.

**FIGURE 4:**
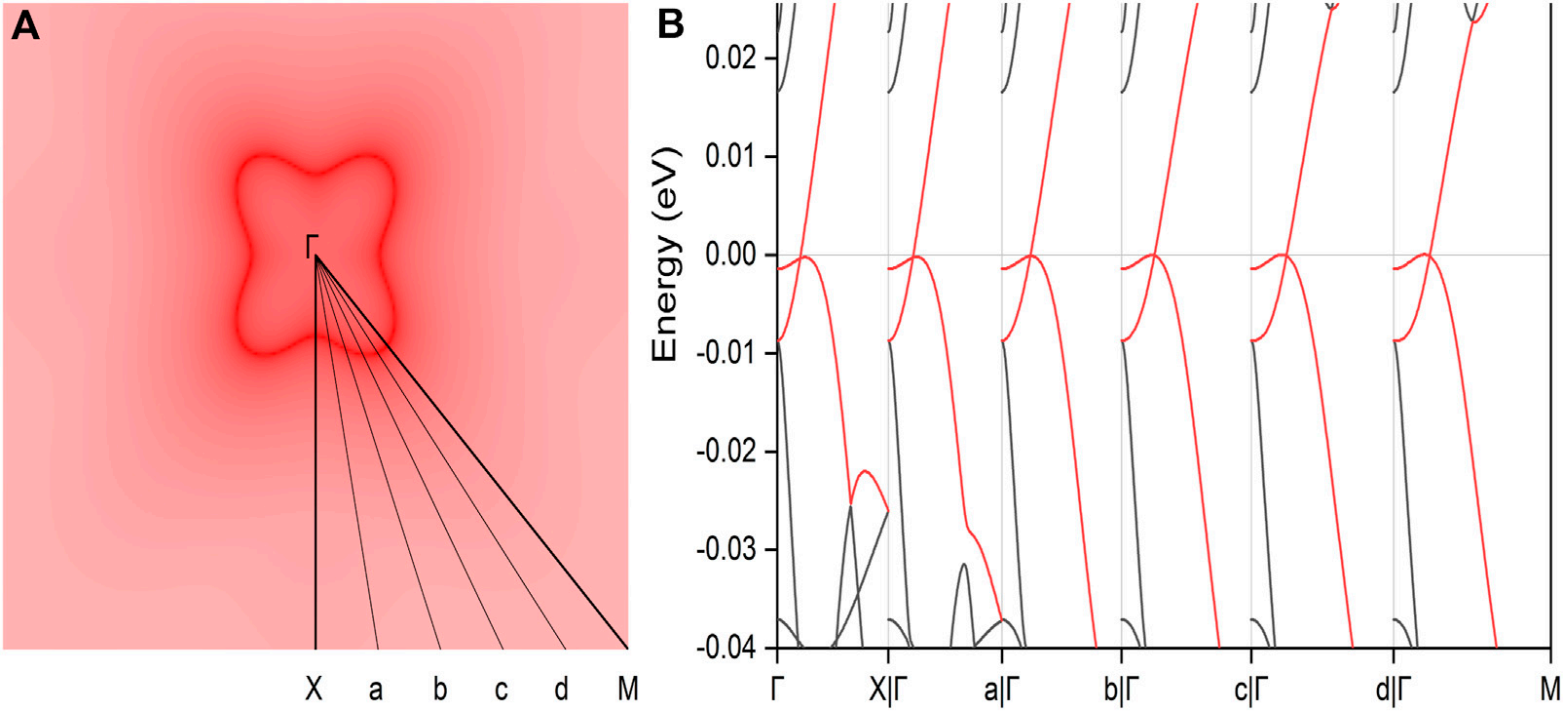
The shape distribution of the nodal loop **(A)** as derived from the subtraction between the corresponding two topological bands and the associated band segments **(B)** along the designated paths traversing the nodal loop, as shown in **(A)**, for the PrClS monolayer.

To elucidate the configuration and geometry of the nodal loop, its profile within the k_z_ = 0 plane is depicted in the left panel of Figure 4. The nodal loop exhibits a unique rounded-square shape, confined to a compact spatial region. To further investigate its electronic properties, a detailed scan of the band segments along the loop was conducted. The resulting band structures, presented in the right panel of Figure 4, reveal all key dispersion characteristics across the entire loop, providing valuable insights into its complex electronic behavior. Significantly, the identification of a complete critical nodal loop in this system represents a major finding, particularly in the context of 2D materials. This discovery reveals a novel structural feature that has not been previously reported in low-dimensional systems. Consequently, it marks a significant advancement in the study of electronic properties in 2D materials, paving the way for new avenues of research and potential applications in advanced material science and condensed matter physics.

**FIGURE 5:**
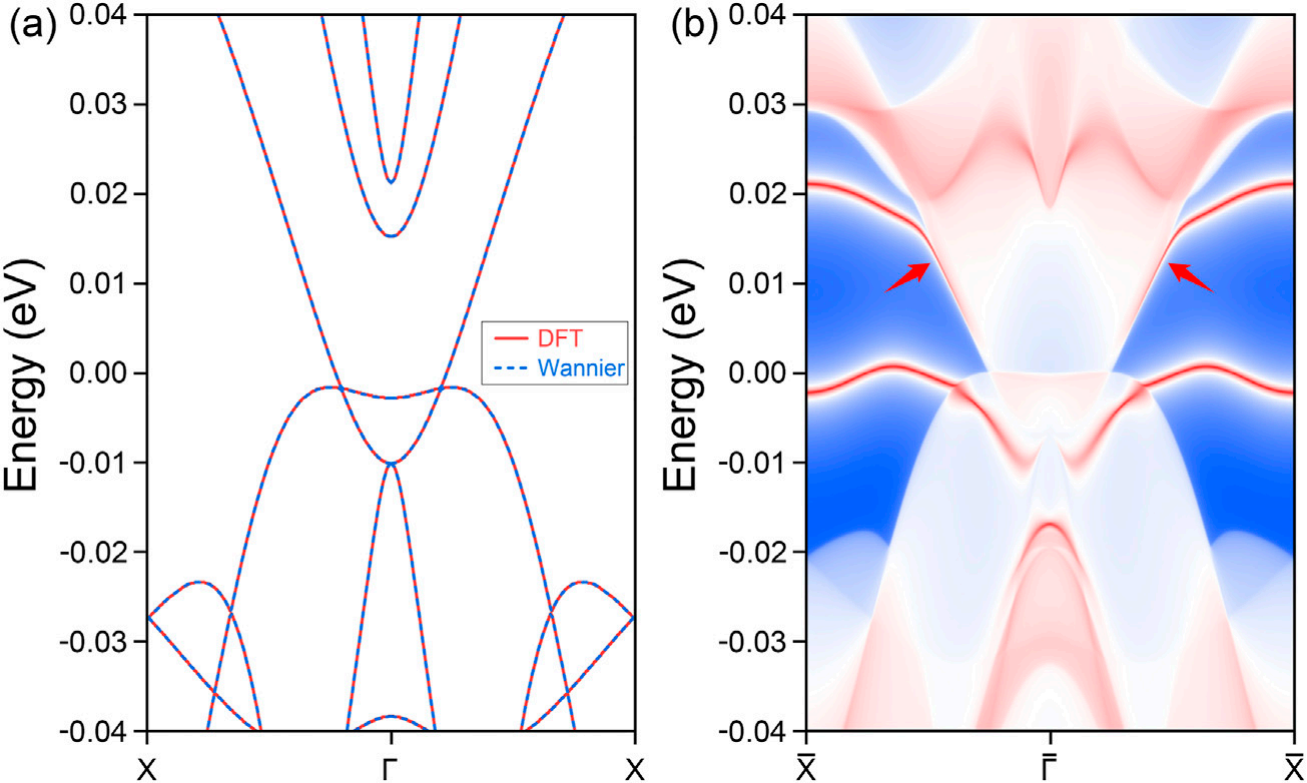
Comparative analysis of the band structure **(A)** for the PrClS monolayer, demonstrating an impeccable concurrence between results from DFT calculations and Wannier function fittings. The corresponding (100) edge state **(B)** along the high-symmetry path $\bar{X}$ – $\bar{\Gamma }$ – $\bar{X}$ .

In 3D materials, topological phases are predominantly defined by the presence of nontrivial surface states. However, when transitioning to 2D systems, these surface states reduce in dimensionality, appearing as edge states. For the spin-up conducting channel of the PrClS monolayer, a detailed orbital analysis reveals that the *f* orbitals of the Pr element play a dominant role in shaping the bands. Based on this orbital composition, a maximally localized Wannier tight-binding Hamiltonian was constructed to explore the material’s topological edge states. The accuracy of the Wannier-based model is confirmed by the excellent agreement between the DFT band structure and the Wannier-fitted band structure, as shown in Figure 5A. Using this model, the topological edge states along the (100) direction were calculated, and the results are presented in Figure 5B. The high-symmetry points along the edge were derived from the surface Brillouin zone, depicted in Figure 1C. The analysis reveals distinct edge states emerging from the nodal loop crossing points, as indicated by the two arrows in the figure, extending toward the boundaries of the Brillouin zone. In addition, arc-like states originating from the nodal point at the Γ point were identified, although these states fall outside the scope of the current study. Due to the compact size of the nodal loop, the corresponding edge states exhibit a significantly extended spatial distribution. Furthermore, these states remain well-separated from the bulk band projection, enhancing their visibility in experimental setups and demonstrating their potential for practical applications. The distinctive properties of these edge states highlight critical topological features, reinforcing the significance of the PrClS monolayer as a promising candidate for advancing 2D magnetic materials research.

**FIGURE 6:**
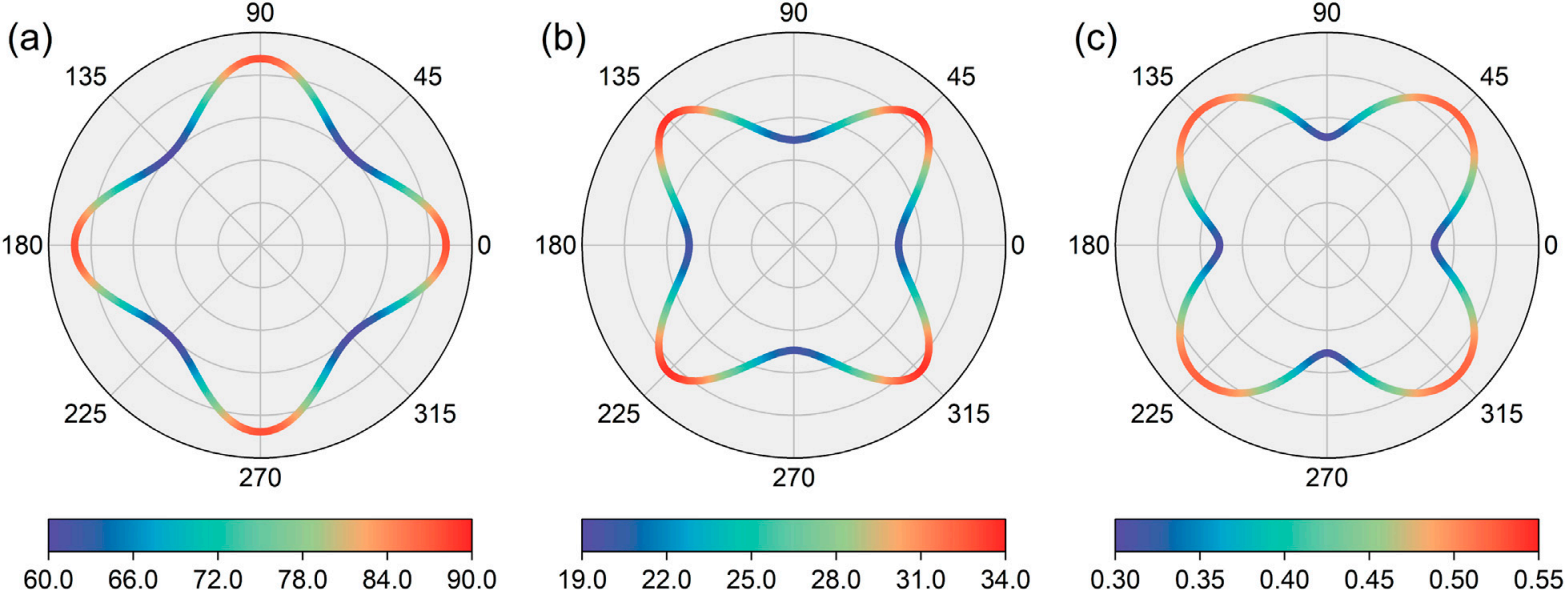
The calculated angular dependence of the Young’s modulus **(A)**, shear modulus **(B)**, and Poisson’s **(C)**, of the PrClS monolayer, where the angle is relative to the x-direction.

To facilitate future experimental characterization and support potential applications, we performed an extensive investigation into the mechanical properties of the PrClS monolayer. Given its 2D tetragonal structure, three independent elastic constants were identified: C_11_ = 96.698 N/m, C_12_ = 29.423 N/m, and C_66_ = 19.784 N/m. According to the elastic stability criteria for tetragonal systems—C_11_ > 0, C_66_ > 0, and C_11_ > |C_12_|—the PrClS monolayer is confirmed to exhibit mechanical stability. Combined with its previously established thermal and dynamic stabilities, this result indicates that synthesizing the PrClS monolayer under experimental conditions is highly feasible. To deepen our understanding of its mechanical behavior, we analyzed its anisotropic properties, focusing on the directional variations in Young’s modulus, shear modulus, and Poisson’s ratio, as illustrated in Figure 6. The analysis reveals pronounced anisotropy. For instance, Young’s modulus reaches its peak along the (100) direction, while the shear modulus and Poisson’s ratio attain their maximum values along the (110) direction. Quantitatively, the calculated anisotropic ratios are 1.457 for Young’s modulus, 1.700 for the shear modulus, and 1.717 for Poisson’s ratio, underscoring substantial directional variations in the material’s mechanical response. These significant anisotropic mechanical properties underscore the crucial role of crystallographic orientation in determining the PrClS monolayer’s behavior, particularly for applications requiring tailored structural configurations or the development of heterojunctions in 2D materials. The strong directional dependence of the mechanical properties highlights the necessity of careful consideration of crystallographic alignment during the design of components for advanced engineering and technological applications.

**FIGURE 7:**
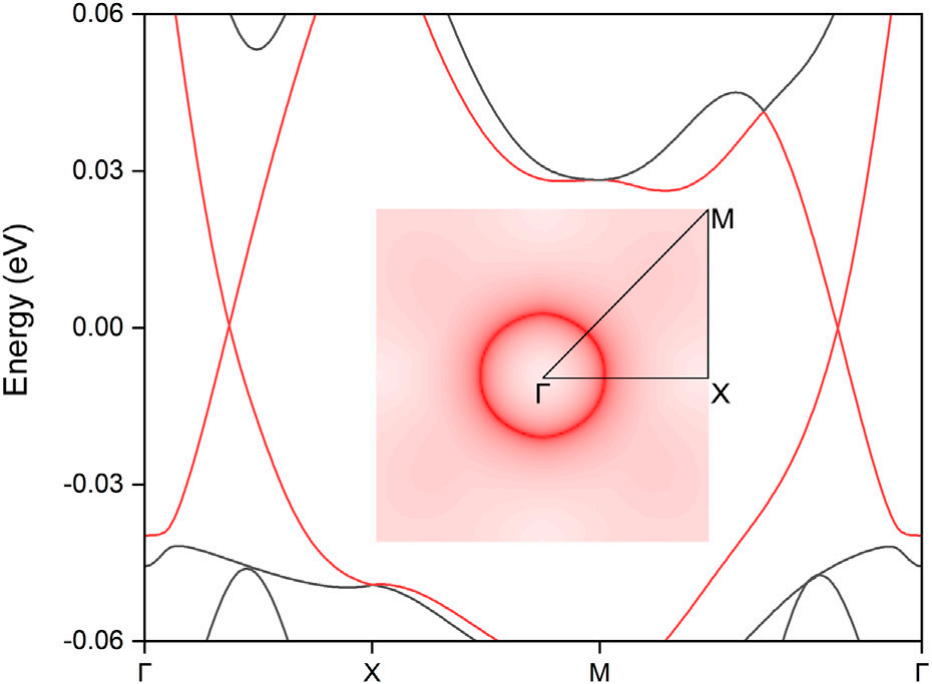
The calculated electronic band structure for the PrClS monolayer under spin-orbit coupling effect. The two topological bands are highlighted in red color. The inset shows the shape distribution of the nodal loop as derived from the subtraction between the corresponding two topological bands.

Finally, the influence of SOC effect, a crucial factor due to the presence of the heavy Pr element, was thoroughly analyzed. SOC often plays a significant role in electronic systems by introducing energy gaps in topological states or altering essential features, potentially disrupting the original topological phase (Moghaddam et al., 2022; Kresse and Furthmüller, 1996; Hafner, 2008; Blöchl, 1994). However, the band structures of the PrClS monolayer under SOC retain their band-crossing conditions, as illustrated in Figure 7. Notably, the inclusion of SOC results in the formation of a circular nodal loop centered around the Γ point, as highlighted in the inset of Figure 7. Furthermore, the dispersion characteristics undergo a transformation from the critical type to an intriguing hourglass shape, which is of particular interest for exploring unconventional electronic behaviors. Detailed analysis of band segment structures reveals that this hourglass dispersion persists along the entire nodal loop, as shown in Supplementary Figure S5 of the supplementary materials. Despite the changes in dispersion type and geometric configuration, the nodal loop remains intact at the Fermi level. The preservation of the nodal loop’s energy alignment at the Fermi level is particularly advantageous, as it not only facilitates precise experimental verification but also enhances its potential utility in spintronics and quantum devices. In addition, the two-dimensional PrClS monolayer exhibits a buckled configuration, which makes it particularly sensitive to strain effects. We conducted a comprehensive analysis of these strain effects, with results detailed in Supplementary Figure S6 of the supplementary materials. Under compressive strain, the nodal ring progressively shrinks and can eventually disappear, demonstrating the critical dependence of the topological features on lattice deformation. Conversely, tensile strain leads to an expansion of the nodal ring, further stabilizing the nodal loop structure. This tunable behavior suggests that choosing a substrate with a lattice mismatch that induces tensile strain could be especially advantageous for fabricating high-performance devices. Such a strategy could enhance the realization of stable and tunable topological states in practical applications.

These findings demonstrate the robust stability and novel properties of the PrClS monolayer, highlighting its potential as a promising candidate for future research in topological materials in both fundamental research and practical applications. A few potential properties and applications stemming from these edge states include: (1) Spin-polarized transport: the presence of magnetic nodal ring edge states with strong spin polarization could lead to highly efficient spin-polarized transport channels. This property could be exploited in spintronic devices, where controlling spin currents independently of charge currents is essential. (2) Quantum anomalous hall effect: the robustness of the magnetic edge states, combined with their potential to generate topologically protected conducting channels, may allow for the realization of the quantum anomalous Hall effect. This phenomenon could be valuable for dissipationless edge state conduction in next-generation electronic devices. (3) Magnetoresistive effects: the interplay between the magnetic nodal ring edge states and external magnetic fields could result in exotic magnetoresistive effects. These could be utilized in high-performance magnetic sensors and memory devices, enabling technologies like magnetic random-access memory with enhanced performance. The combination of unique structural, electronic, and topological features not only strengthens its utility in practical applications but also inspires further advancements in condensed matter physics and materials science. The PrClS monolayer proves to be an exceptional system capable of fostering innovative applications while advancing theoretical concepts in the exploration of topological materials and their associated phenomena.

## Conclusion

This study introduces and extensively investigates the 2D material PrClS, highlighting its unique physical and electronic properties. Under its ferromagnetic ground state, the PrClS monolayer demonstrates a half-metallic nature with 100% spin polarization of conducting electrons in the spin-up channel. Notably, in the spin-up direction, two electronic bands form a closed nodal loop, which exhibits a critical dispersion type and is precisely aligned with the Fermi energy level. The symmetry characteristics of this nodal loop and the detailed band dispersion configuration have been thoroughly analyzed and presented. Using a maximally localized Wannier tight-binding Hamiltonian, we further investigated the topological edge spectrum of PrClS. Our results reveal the emergence of two distinct edge states, originating from the crossing points of the nodal loop. These edge states are well separated from the bulk band projection while maintaining a significant spatial distribution, which enhances their experimental detectability. When the SOC effect is taken into account, the nodal loop crossings persist but transform into an intriguing hourglass-like dispersion. This resilience of the nodal loop against SOC perturbations underscores its robustness, making it a promising candidate for experimental validation and future implementation in advanced technologies. To support potential environmental and practical applications, the mechanical properties of PrClS were comprehensively evaluated. Key parameters such as Young’s modulus, shear modulus, and Poisson’s ratio were derived, with their anisotropic behaviors analyzed in detail. The results demonstrate significant mechanical anisotropy, emphasizing the importance of aligning the material’s crystallographic orientation carefully in engineering designs and technological applications. In summary, the PrClS monolayer, with its remarkable combination of half-metallicity, spin-polarized nodal loop states, and robust topological properties, represents an ideal platform for exploring fundamental physics in 2D magnetic systems. This study provides a foundation for future investigations and practical applications involving spintronics, topological materials, and mechanically anisotropic behavior in low-dimensional materials.

## Data Availability

The raw data supporting the conclusions of this article will be made available by the authors, without undue reservation.
