# Supplementary material for: Spin polarized nodal loop state at Fermi level in the monolayer PrClS
Source: Front Chem. 2025 Feb 27;13:1544147. doi: 10.3389/fchem.2025.1544147 (PMC11903714; doi:10.3389/fchem.2025.1544147)
Supplement: Supplementary file 1 [file Table1.DOCX]

# Spin polarized nodal loop state at Fermi level in the monolayer PrClS

Fang Fang^1,2^, Yanwei Yu^1^, Li Zhang^3,*^ and Yang Li^1,2,*^

^1^ The Engineering and Technology Research Center of Myocardial Prevention and Rehabilitation, The Fourth Medical College of Harbin Medical University, Harbin, China.

^2^ CTOF-PET/CT/MR Center, The Fourth Medical College of Harbin Medical University, Harbin, China.

^3^ College of Mechanics, Changchun Institute of Technology, Changchun, China.

^*^ Emails: doctoryangli@hrbmu.edu.cn and [lizhang@ccit.edu.cn](mailto:lizhang@ccit.edu.cn)


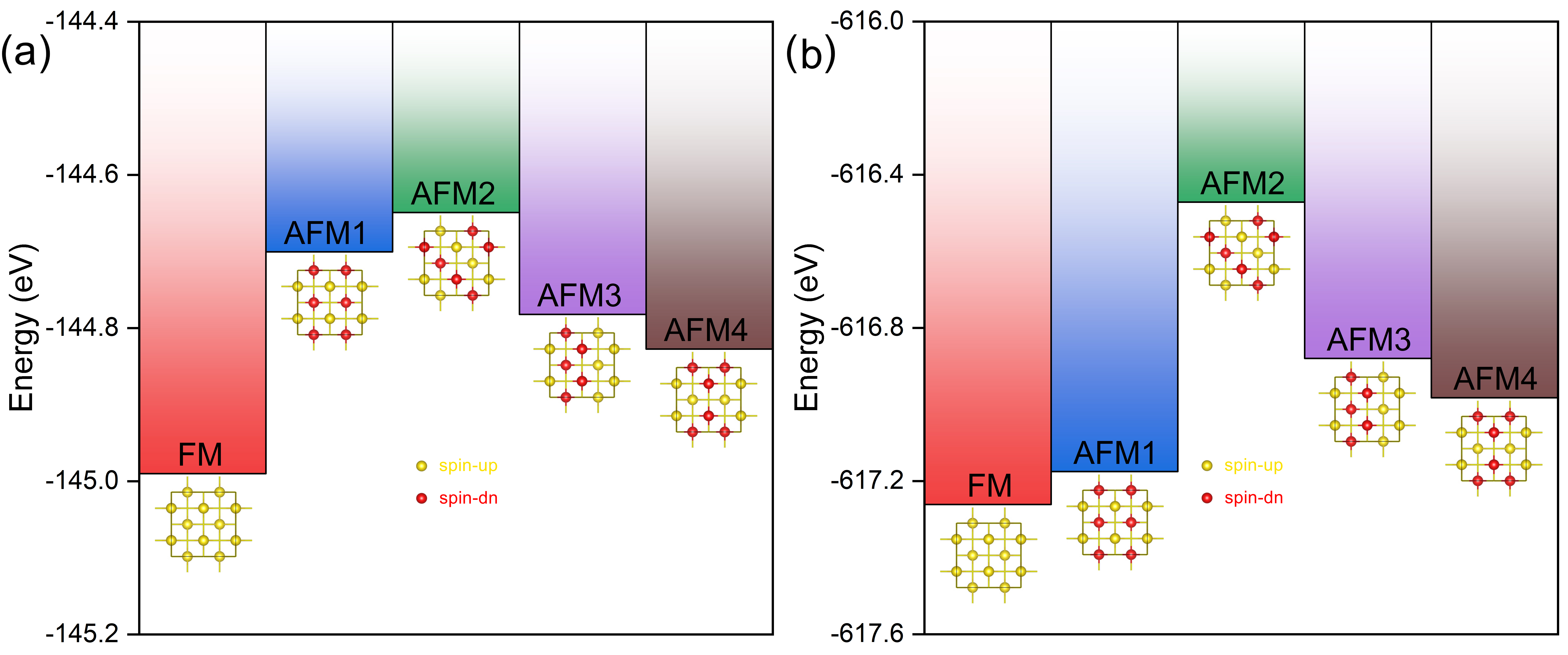


Figure S1: Comparative total energy analysis of magnetic configurations using (a) PBE-GGA formalism and (b) SCAN meta-GGA functional. The magnetic configurations are systematically explored within a 2×2 supercell, with detailed magnetic arrangement schematics provided in the corresponding inset illustrations.

To address the material's thermal stability and estimate its Curie temperature, we performed Monte Carlo simulations based on the Ising model, employing the Metropolis algorithm. For these simulations, we considered magnetic interactions up to the third-nearest neighbors, and the system’s Hamiltonian was expressed as:

$$\text{H}\text{=}-\sum_{\text{i}\text{j}} \text{J}_{\text{1}}\text{M}_{\text{i}}\text{M}_{\text{j}}-\sum_{\text{k}\text{l}} \text{J}_{\text{2}}\text{M}_{\text{k}}\text{M}_{\text{l}}-\sum_{\text{m}\text{n}} \text{J}_{\text{3}}\text{M}_{\text{m}}\text{M}_{\text{n}}$$

where $\text{J}_{\text{1}}$, $\text{J}_{\text{2}}$, and $\text{J}_{\text{3}}$ are the coupling parameters for magnetic ion pairs corresponding to first, second, and third-nearest neighbor interactions, respectively; $\text{M}_{\text{i}}$ represents the magnetic moment at site i; and (ij), (kl), and (mn) denote the first, second, and third nearest neighbor pairs. These coupling parameters were derived from the energy differences between the FM and AFM configurations calculated using DFT. The determined values are summarized in the above Table S1. To reduce the effects of periodic boundary constraints, we constructed a 32×32 supercell of the PrClS monolayer for Monte Carlo simulations. At each temperature step, the first 1×10^5^ iterations were used for thermal equilibration, and the subsequent 1×105 steps were averaged to calculate key physical quantities. The evolution of magnetization and specific heat capacity as functions of temperature is presented in the above Figure 3. A sudden drop in the average magnetic moment near 400 K indicates the phase transition from a ferromagnetic to a paramagnetic state. Using the magnetic specific heat, the precise Curie temperature of the monolayer PrClS was determined to be 416.85 K. This remarkably high Curie temperature, exceeding room temperature, highlights the exceptional potential of PrClS for spintronic applications operating above room temperature.

Table 1 The calculated magnetic coupling parameters for the monolayer PrClS.

| Compound | J_1_/meV | J_2_/meV | J_3_/meV |
| --- | --- | --- | --- |
| PrClS | 2.133 | 1.441 | 0.259 |


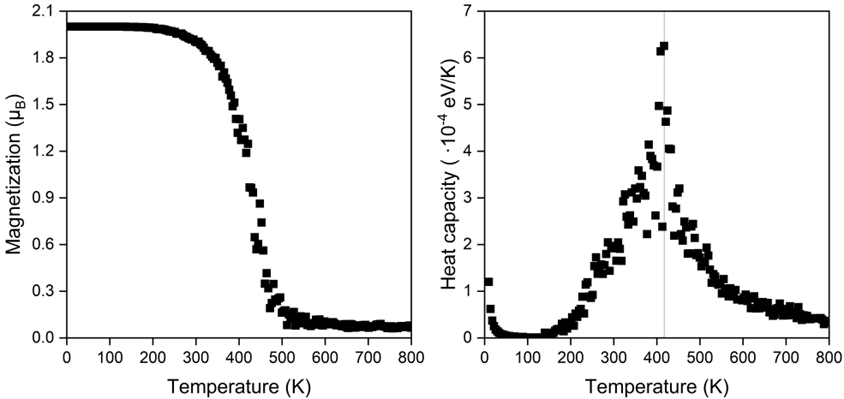


Figure S2: Monte Caro simulation results of the temperature dependent magnetization and specific heat capacity for the monolayer PrClS compound. The vertical dashed line indicates the position of the Curie temperature as 416.85 K.


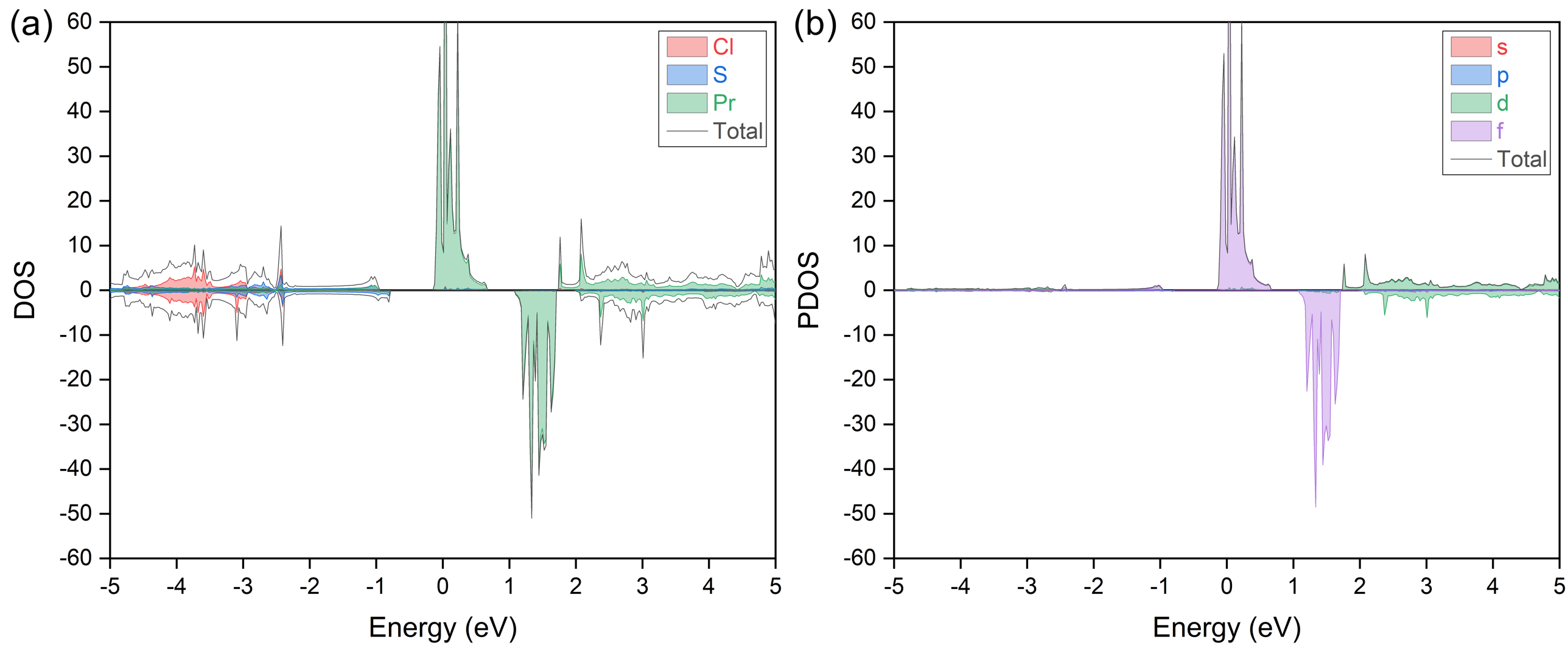


Figure S3: Total Density of States (DOS) for monolayer PrClS, highlighting contributions from individual elements. (b) Projected Density of States (PDOS) for the Pr element, detailing contributions from specific orbitals. The positive values of DOS/PDOS represent the spin-up channel, while negative values indicate the spin-down channel.


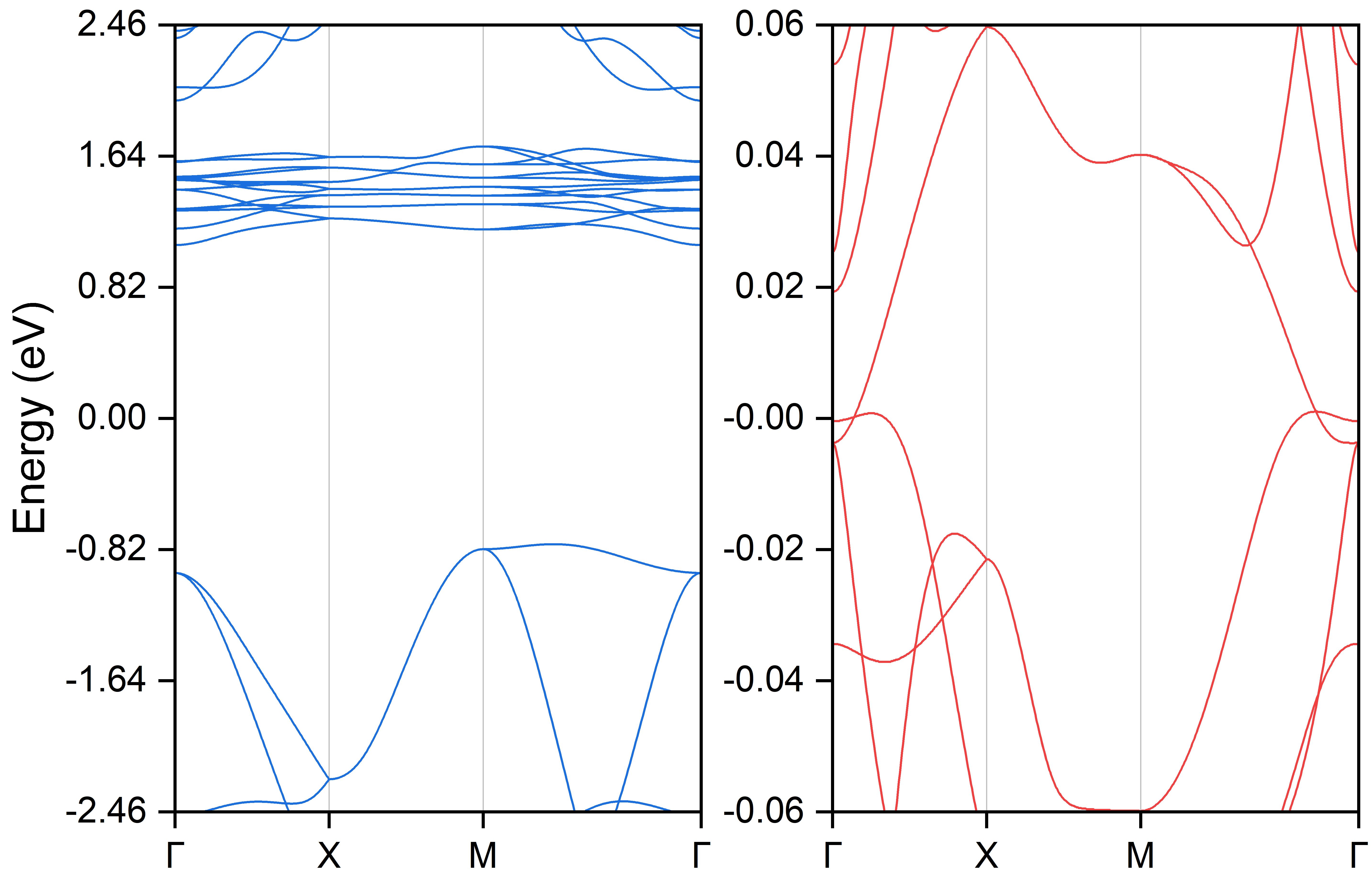


Figure S4: Electronic band structures of the PrClS monolayer under SCAN meta-GGA functional. The spin-down band structure exhibits an insulating character with a band gap, while the spin-up band structure displays metallic behavior, featuring two band crossing points at the Fermi level.


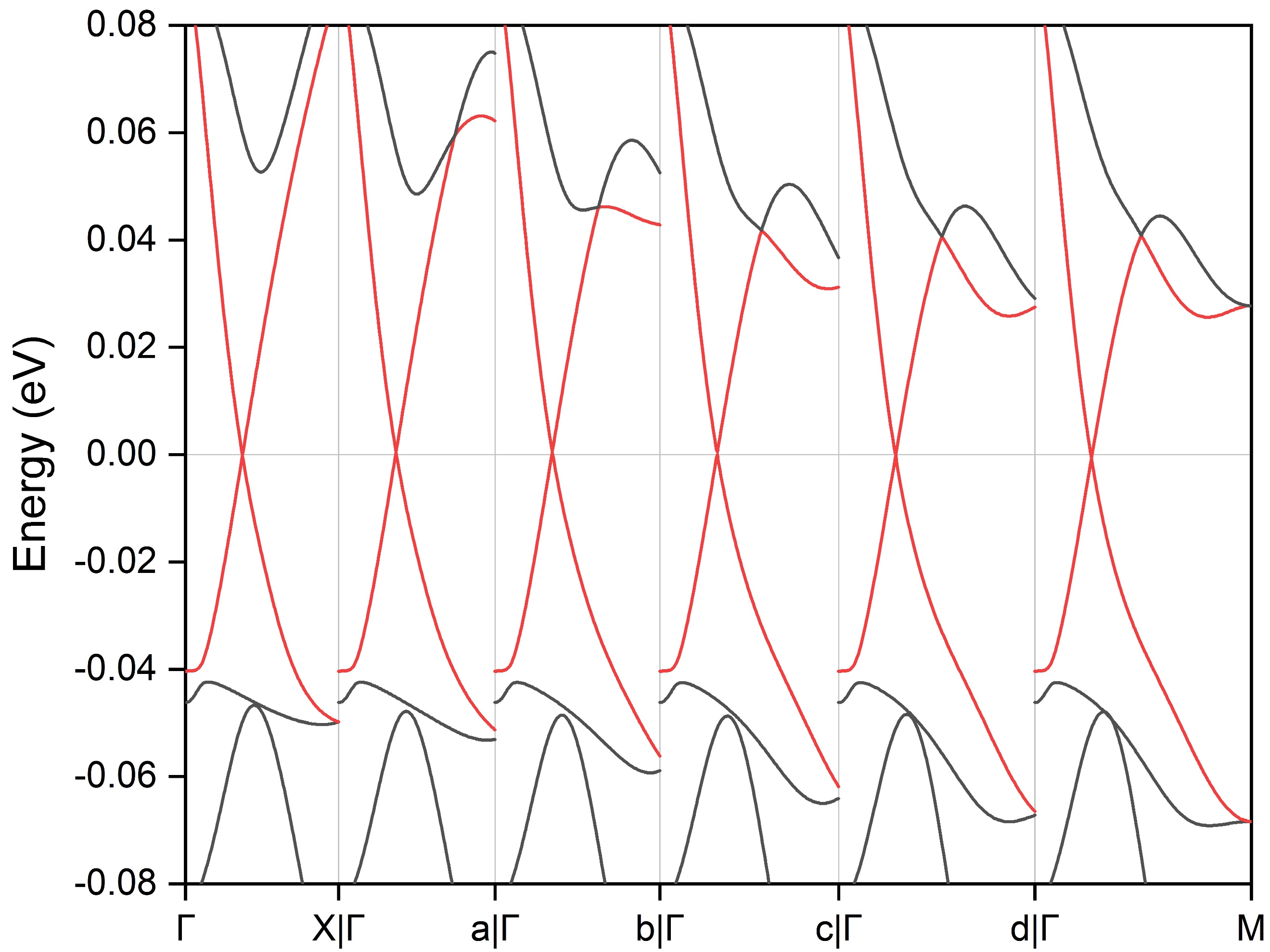


Figure S5: The calculated band segments along the various paths traversing the nodal loop, as shown in Figure 5(a), for the PrClS monolayer under spin-orbit coupling effect. The two topological bands are highlighted in red color.


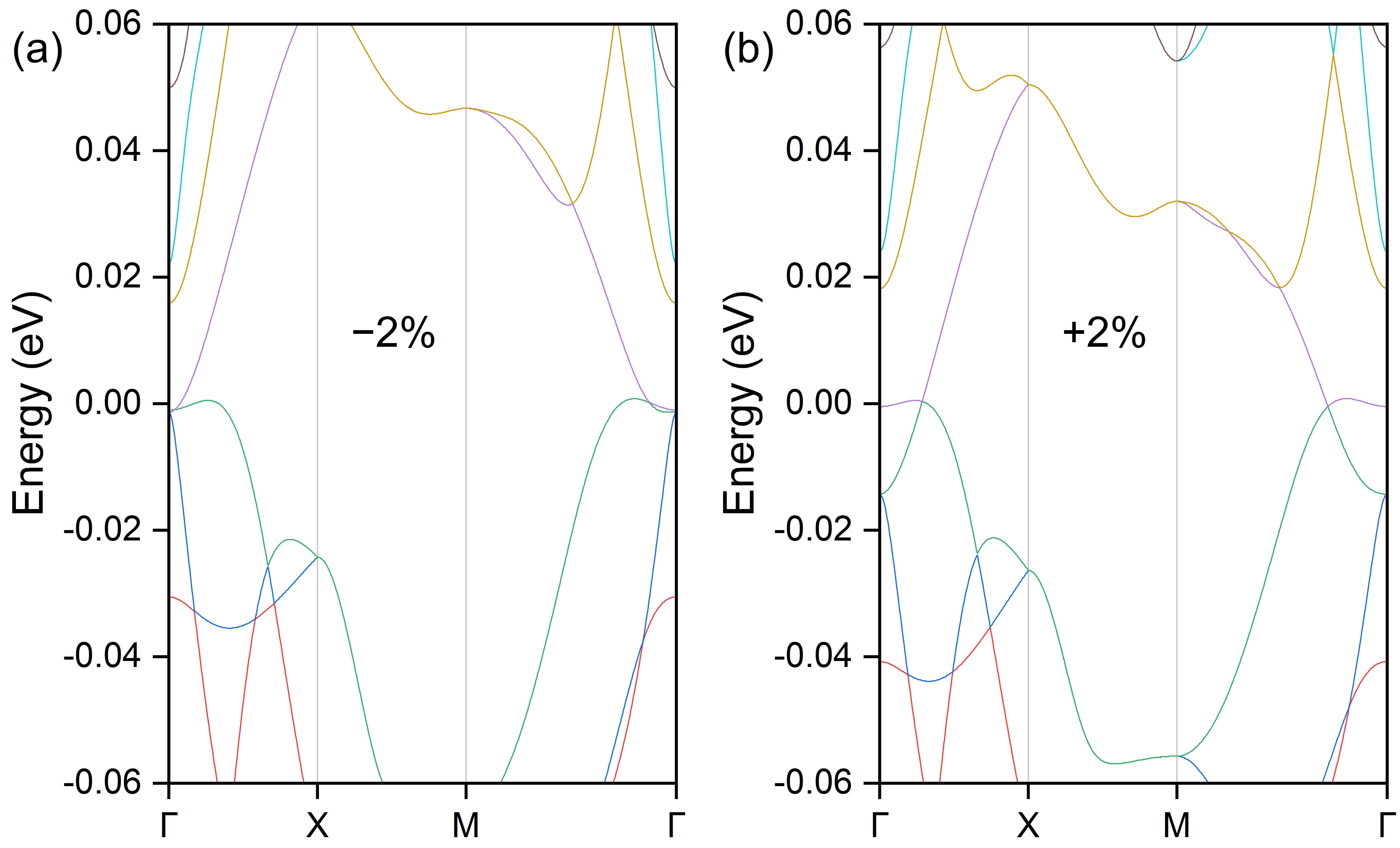


Figure S5: Strain condition on the electronic band structures of the PrClS monolayer solo in the spin-up channel. Under tensile strain, the topological crossing is even further expanded.
